# New waves underneath the purple strain

**DOI:** 10.1111/1751-7915.12409

**Published:** 2016-08-30

**Authors:** Marta Tortajada

**Affiliations:** ^1^ BIOPOLIS S.L. Parc Científic Universitat de València. C/Catedrático Agustín Escardino, 9 Paterna Valencia 46980 Spain

## Abstract

Successful merging of chemical and biotechnological operations is essential to achieve cost‐efficient industrialization of bio‐based processes. The demonstration of the use of syngas, derived from microwave assisted pyrolysis of municipal solid waste, for the improved growth and poly‐3‐hydroxybutyrate production in *Rhodospirillium rubrum*, stands out as an example of the synergistic contribution of chemical engineering and applied microbiology to sustainable biomaterial manufacturing, paving the way to similar applications for other syngas derived bioproducts.

Great efforts have been devoted in the past decades to develop biotechnology‐based factories, so called biorefineries, which convert renewable raw materials into valuable chemicals. In a fine combination of chemical engineering and applied microbiology, Revelles *et al*. (2016) validate the use of syngas, derived from microwave assisted pyrolysis of municipal solid waste (MSW), for *Rhodospirillium rubrum* growth and production of poly‐3‐hydroxybutyrate (PHB), a reference bio‐based and biodegradable plastic (Zinn *et al*., 2001).

In contrast to plant oils, grains and sugars used as fermentation substrates in first generation refineries, residual biomass‐rich matrixes, such as MSW or sewage sludge, bear great potential as feedstocks for second generation plants, thanks to their generalized availability, vast surplus and food‐chain independent origin. However, direct processing is hindered by their intrinsic complex composition, reduced carbon content and recalcitrance to hydrolysis. Syngas (CO+H_2_) obtained by thermochemical conversion of these wastes, offers the advantage of yielding homogeneous composition and higher carbon source concentration (Drzyzga *et al*., 2015).

Previous contributions have shown how microwave assisted pyrolysis (MIP) for syngas production results in reduced waste volumes, rapid and selective heating and improved quality of the final products in comparison to conventional pyrolysis, together with portable and cost‐efficient processes for *in situ* treatment of waste (Beneroso *et al*., 2014, 2015). Revelles *et al*. (2016) effectively utilize this stream for the first time with *R. rubrum* cultures for biomass and biodegradable plastic synthesis. From the present results, noteworthy is the fact that MIP syngas is not only consumed faster than synthetic syngas (1.3 times more in light, and twice as fast in darkness) but also provides increased yields on biomass, demonstrating a more efficient assimilation of the carbon fraction in the gas effluent.

*Rhodospirillium rubrum* is a versatile non‐sulphur bacterium that can grow in aerobic or anaerobic conditions, the latter allowing the expression of photosynthetic pathways resulting in its distinct purple‐red colour. Anaerobic growth also enables CO and CO_2_ assimilation, both in light and darkness. The ability of *R. rubrum* for producing PHB when grown on syngas has been described (Do *et al*., 2007; Choi *et al*., 2010), and makes this singular bacterium, together with other syngas‐converters such as *Clostridium ljungdahlii* (Köpke *et al*., 2010; Schiel‐Bengelsdorf and Dürre, 2012), a valuable biocatalyst for third generation, gas based biorefineries.

Results from Revelles *et al*. (2016) demonstrate that *R. rubrum* can readily use synthetic or MIP syngas, in a broad range of compositions and operational conditions, light or darkness. In this way, syngas streams with lower CO and CO_2_ content can also be assimilated into biomass and long‐chain hydrocarbons. Interestingly, low molecular weight hydrocarbons, found as minor impurities in MIP syngas, do not adversely affect bacterial growth either. The adaptability to variations in feedstock composition, in terms of lower carbon substrate concentrations and resistance to impurities, is shared by other gas‐fed systems, such as the conversion of methane from biogas streams by methanotrophic cultures, again for biomass and PHB synthesis (del Cerro *et al*., 2012; Rostkowski *et al*., 2012). In comparison to the chemically catalysed syngas conversion into higher molecular weight compounds, as in the Fischer‐Tropsch process, and apart from obvious lower energy requirements, the amenable and resilient character of *R. rubrum* as a biocatalyst is the basis of a major competitive advantage of the overall process.

The creation of PHB out of cheap and widely available renewable resources, such as syngas, is a major and critical step to ultimately launch these ever‐promising materials towards massive commercial production (Chen, 2009). Indeed, it is well known that raw materials contribution accounts for more than a third of operational costs in PHB manufacturing (Solaiman *et al*., 2006). Syngas fermentation stands out as a breakthrough technology for the cost‐effective generation of chemicals and fuels from a wide range of low‐cost feedstocks, including MSW, but also industrial waste, agricultural biomass and industrial off‐gases. Syngas fermentation has already been demonstrated at commercial scale for ethanol, and is underway for other building blocks, such as 2,3‐butanediol (Köpke *et al*., 2011). In this context, the fact that Revelles *et al*. (2016) achieve production of PHB from microwave syngas is particularly relevant: since microwave assisted pyrolysis overcomes limitations of conventional pyrolysis (Fernández *et al*., 2011), it seems reasonable to expect that MIP syngas fermentation benefits from an enhanced overall process efficiency. Most probably, MIP syngas is also a worthy substrate for other relevant syngas‐based bioprocesses, as the ones involving the Wood‐Ljungdahl pathway (Köpke *et al*., 2010). Therefore, the validation by Revelles *et al*. (2016) of the usefulness of MIP syngas as a fermentation substrate for PHB production opens the door to similar applications for other syngas derived bioproducts.

Undoubtedly, both strain and process development strategies will further promote this approach. Synthetic biology and omics tools are being intensively exploited to expand the knowledge base on *R. rubrum* and to increase PHB productivity by remodelling bacterial metabolism (Jin and Nikolau, 2012; Heinrich *et al*., 2015; Klask *et al*., 2015; Revelles *et al*., 2016). An intrinsic limitation of the process, substrate solubility, may be overcome with novel reactor configurations and feeding strategies (Munasinghe and Khanal, 2010). In this sense, the Revelles *et al*. (2016) contribution paves the way for improved PHB production using syngas from MSW, as an example of sustainable biomaterial manufacturing through biotechnology.

In conclusion, novel chemical and biotechnological processes are required to bring cost‐efficient bio‐based plants to industrial reality. The success of such hybrid biorefineries will stand on the synergistic contribution of both chemical and fermentative processes. Revelles *et al*. (2016) demonstrate in this article that emerging chemical processing technologies, such as microwave assisted pyrolysis, can supply valuable substrates for existing biorefinery platforms. At least as importantly, versatile *R. rubrum* is shown to integrate the fluctuations in syngas composition to constant conversion, becoming a tuning‐filter for biomass and biomaterial production. May there be many more such new applications for this and other (old) bacteria.
